# Routine parallel diagnosis of malaria using microscopy and the malaria rapid diagnostic test SD 05FK60: the experience of Médecins Sans Frontières in Myanmar

**DOI:** 10.1186/1475-2875-12-167

**Published:** 2013-05-21

**Authors:** Cara S Kosack, Wint Thu Naing, Erwan Piriou, Leslie Shanks

**Affiliations:** 1 Médecins Sans Frontières International, Plantage Middenlaan 14, 1018 DD Amsterdam, The Netherlands; 2 Médecins Sans Frontières Holland, Yangon, Myanmar; 3 Médecins Sans Frontières Holland, Amsterdam, The Netherlands

**Keywords:** Malaria, RDT, Rapid diagnostic test, Sensitivity, Specificity, Diagnostic test accuracy, Parasite lactate dehydrogenase tests, *Plasmodium vivax*, *Plasmodium falciparum*, SD 05FK60

## Abstract

**Background:**

Malaria rapid diagnostic tests (RDTs) are commonly used in Médecins Sans Frontières (MSF) programmes to detect acute malaria infection. Programmes in regions with both *Plasmodium falciparum* and non-falciparum malaria (i.e. *Plasmodium ovale*, *Plasmodium malariae* and *Plasmodium vivax*) use a three-band *P. falciparum*/Pan test such as the SD Bioline Malaria Ag P.f*/*Pan 05FK60 (Standard Diagnostics, Kyonggi, Republic of Korea), hereafter referred to as SD 05FK60, as used by the MSF-Holland clinics in Rakhine state, Myanmar. In spite of published reports of generally good test performance, medical and paramedical staff on the ground often doubt the diagnostic accuracy of these RDTs.

**Methods:**

Parallel testing with malaria microscopy and RDT was conducted at two clinics in Rakhine state, Myanmar, for a period of 14 months as a programmatic response due to doubts and concerns of medical and paramedical staff into malaria RDTs.

**Results:**

A total of 2,585 blood samples from non-pregnant suspected malaria patients were examined by the SD 05FK60 RDT and microscopy at two clinics in Myanmar from October 2010 to December 2011. The reference standard microscopy diagnosed 531 *P. falciparum* and 587 *P. vivax* or *P. malariae* mono-infections. The overall sensitivity for *P. falciparum* detection by the SD 05FK60 was 90.2% (95% CI: 87.4-92.6) and for *P. vivax*/*P. malariae* 79.4% (95% CI: 75.9-82.6). The overall specificity for *P. falciparum* detection by the SD 05FK60 was 98.5% (95% CI: 97.7-99.1) and for *P. vivax*/*P. malariae* 98.7% (95% CI: 97.9-99.2). The sensitivity for *P. falciparum* was >91% for parasitaemia levels of >100-1,000 parasites/μl and increased for *P. vivax*/*P. malariae* with the parasitaemia level but was overall lower than for *P. falciparum* 25/408 and 13/420 cases, respectively, of *P. falciparum* and non-falciparum malaria were missed by the RDT.

**Conclusion:**

In field conditions in Myanmar, the SD 05FK60 malaria RDT performed consistent with other reports. The test detected malaria caused by *P. vivax*/*P. malariae* to a lesser extent than *P. falciparum* infection. Sensitivity improved with increasing parasitaemia level, however even at higher levels some infections were missed. The SD 05FK60 is adequate for use in settings where high quality microscopy is not available.

## Background

Microscopy is the reference standard for diagnosing malaria but is time consuming and requires considerable training and experience. It is ill suited as a diagnostic tool in settings where the case load is high and resources limited [1,2]. Lateral-flow immunoassays (LFIs), often called rapid diagnostic tests (RDTs), are an attractive alternative as they are quick and easy to carry out [1-7]. In practice however, malaria is often diagnosed solely on the basis of clinical symptoms, without using either method; this results in over-treatment of malaria and the risk of missing true underlying pathologies. To improve the accuracy of malaria diagnosis and avoid unnecessary treatment, the World Health Organization (WHO) recommends parasitological confirmation of malaria whenever possible, by RDT or by microscopy [1,8-11].

Among the newer generation of malaria RDTs are three-band tests that detect both the *Plasmodium falciparum*-specific antigen histidine-rich protein 2 (HRP2) and the pan-*Plasmodium*-specific lactate dehydrogenase (pLDH), expressed in the four most common malaria parasite species [*P. falciparum*, *Plasmodium ovale, Plasmodium malariae* and *Plasmodium vivax*[6,12-16].

In 2009, Médecins Sans Frontières (MSF) introduced routine use of HRP2/pan-pLDH RDTs into its programmes at sites where both *P. falciparum* and non-falciparum malaria are present (i e, Southeast Asia, the Americas and the Ethiopian highlands). In Myanmar, which has an enormous malaria burden, accounting for 7% of all malaria cases in Southeast Asia and India [7], *P. vivax* is second only to *P. falciparum* as the cause of malaria, so the test chosen was the SD Bioline 05FK60 Malaria Ag P.f*/*Pan (Standard Diagnostics, Kyonggi, Republic of Korea), hereafter referred to as SD 05FK60.

The choice was made for the SD 05FK60 because it was one of the best performing assays in the WHO/TDR (Special Programme for Research and Training in Tropical Diseases)/FIND (Foundation for Innovative New Diagnostics)/CDC (Centers for Disease Control) evaluation in Round 1 and because continuous supply and importation to Myanmar could be guaranteed. However, since 2007, evaluations of the SD 05FK60 were conducted in returned travellers or as mentioned before by the WHO/TDR/FIND/CDC programme but not on a field site. In these evaluations, the SD 05FK60 showed good performance for detection of malaria caused by *P. falciparum,* with varying results for *P. vivax* detection [17-22].

However, in spite of the generally good performance of malaria RDTs, their use on the ground can be problematic: medical staff frequently mistrust the results and problems with the products (e g, poor migration of blood and buffer, faint lines, incorrect placement of the lateral-flow strip in the cassette) are encountered regularly [23-25]. This often results in low coverage of RDT implementation and poor adherence to RDT results (as the basis for treatment decisions) in clinical practice [26]. In order to monitor the performance of this newly introduced RDT in MSF’s programmes in Myanmar and to respond to clinicians’ concerns of poor RDT performance, it was decided to test patients in parallel with microscopy and RDT until the end of 2011 in our programmes in Rakhine State. In Myanmar, approximately 68% of the population of over 60 million total, is thought to be at risk for malaria with the high risk areas concentrated near international borders, including Rakhine state. The yearly burden in the entire country is to be over two million cases [16].

## Methods

### Investigation sites and procedures

MSF has been working in Myanmar’s Rakhine State since 1993. Primary health care (PHC), including the diagnosis and treatment of malaria, is provided free of charge at two clinics, in Sittwe and Thetkalpyin, located in the eastern region of Rakhine. All patients presenting at the Sittwe and Thetkalpyin clinics with fever or a history of fever in the prior 24 hours were immediately tested for malaria on capillary blood with the SD 05FK60 RDT. In addition, a slide was prepared from the same capillary sample and examined separately in the laboratory regardless of the SD 05FK60 result. The laboratory technician was not aware of the SD 05FK60 result when examining the smear. Pregnant women screened for malaria as part of antenatal care received the same procedure but were not included in this analysis.

This routine parallel testing of the described population was carried out from 25 October, 2010 until 23 December, 2011 with one month of data lost (23 February to 28 March 2011) as a result of security incidents in eastern Rakhine State.

### Microscopy

Thick and thin blood films were prepared and stained with 10% Giemsa solution (pH 7.2) for 10 min and examined by light microscopy using x1,000 magnification. At least 200 fields had to be examined before designating a sample ‘negative’, or more accurately, ‘no malaria parasites seen’. Positive findings were graded on the thick smear using the ‘plus’ system scale: + (1 to 9 trophozoites in 100 fields); ++ (1 to 10 trophozoites in 10 fields); +++ (1 to 10 trophozoites per field); ++++ (>10 trophozoites per field). These scores were used to estimate parasite densities: + = 10 to 90 parasites/μl; ++ = 100 to 1,000 parasites/μl, +++ = 1,000 to 10,000 parasites/μl; ++++ = >10,000 parasites/μl, assuming a white blood cell count of 8,000/μl. The species was identified using the thin smear.

### Rapid diagnostic test

The SD 05FK60 RDT is a three-band lateral-flow immunochromatographic antigen detection test in a cassette format and testing was carried out according to the manufacturer’s instructions. Readings were taken in daylight, assisted by standard electric lighting. A negative result is indicated by the presence of a single line, the ‘C’ control line, in the result window. A *P. falciparum*-positive result was indicated by presence of two colour bands, the *P. falciparum* test line and the ‘C’ control line. The presence of the ‘Pan’ test line and the ‘C’ control line (again, two colour bands) indicated a *P. vivax, P. ovale.* or *P. malariae*-positive result or a mixed non-falciparum infection. The presence of three colour bands, the ‘*P. falciparum*’ and ‘Pan’ test lines and the ‘C’ control line indicates a *P. falciparum-*positive result or a mixed infection. In cases where the control line did not appear, the results were interpreted as invalid and the test repeated with a new device.

### Statistical analysis

Data were entered in an Excel file and analysed using Stata 11.0 statistical software (Stata Corporation, College Station, Texas, USA). Samples with pure gametocytaemia were considered negative. Microscopically identified mixed infections were considered separately (Table 1) and not included in the calculations of test accuracy (Table 2). Sensitivity, specificity and predictive values were calculated with 95% confidence intervals (CI) for the detection of *P. falciparum* and non-falciparum species separately. When calculating the performance parameters for *P. falciparum* all negative samples plus all *P. falciparum* positive samples by microscopy contributed to the analysis. Similarly for the non-falciparum analysis all negative samples and all non-falciparum positive samples by microscopy contributed to the analysis. Likelihood ratios were calculated using the following formulae: LR + = sensitivity/(1–specificity) and LR– = (1–specificity)/sensitivity (Table 2).

**Table 1:** **Malaria diagnosis: microscopy compared with rapid diagnostic test SD Bioline Malaria Ag P.f*/*Pan 05FK60**

		**SD Bioline Malaria Ag P.f/Pan (05FK60)**			
**Microscopy result**	**No. of samples**	**Negative (%)**	***P. falciparum *(%)**	Pan* (%)	**P.f/Pan (%)**
		**(control band only)**	**(*P. falciparum *band only)**	**(Pan band only)**	**(*P.f *and pan band)**
No parasites seen	1,337	1,306 (97.7)	13 (1.0)	11 (0.8)	7 (0.5)
*P. falciparum*	531	39 (7.3)	162 (30.5)	13 (2.5)	317 (59.7)
*P. vivax* or *P. malariae*	587	118 (20.1)	3 (0.5)	454 (77.3)	12 (2.0)
Mixed P.f/pan	130	0 (0.0)	6 (4.6)	2 (1.5)	122 (93.9)

A total of 2,585 blood samples was examined at the Sittwe and Thetkalpyin clinics, Rakhine State, Myanmar.

*SD 05FK60 detects the pan-*Plasmodium-*specific lactate dehydrogenase (pLDH) expressed in *P. falciparum, P. vivax, P. ovale* and *P. malariae*).

**Table 2:** Performance of SD Bioline Malaria Ag P.f/Pan (05FK60) rapid diagnostic test (mixed infections excluded)

**Microscopy results**	***P. falciparum***	***P. vivax/P. malariae***
	**N = 1,868 (531 positive)**	**N = 1,924 (587 positive)**
**Sensitivity (95% CI)**	90.2 (87.4-92.6)	79.4 (75.9-82.6)
**Specificity (95% CI)**	98.5 (97.7-99.1)	98.7 (97.9-99.2)
**PPV (95% CI)**	96.0 (93.9-97.5)	96.3 (94.2-97.8)
**NPV (95% CI)**	96.2 (95.0-97.2)	91.6 (90.0-93.0)
**LR + (95% CI)**	60.3 (39–93.2)	59.0 (37.2-93.5)
**LR- (95% CI)**	0.10 (0.08-0.13)	0.21 (0.18-0.25)

A total of 2,585 blood samples was examined at the Sittwe and Thetkalpyin clinics, Rakhine State, Myanmar.

PPV: positive predicted value; NPV: negative predictive value; LR: likelihood ratio; LR+: sensitivity/(1–specificity); LR–: (1–specificity)/sensitivity.

### Quality control

All malaria RDTs purchased by MSF must pass the WHO lot-testing programme before being shipped to field sites [27]. In addition, the programme follows the WHO Malaria Microscopy Quality Assurance recommendation [28]. In accordance with this protocol, slides were sent to the Shoklo Malaria Research Unit (SMRU) in Mae Sot, Thailand, for external quality control.

### Ethical statement

This manuscript reports a descriptive retrospective analysis of routinely collected data, and therefore no protocol was submitted for ethical review. Data collected contained no personal identifiers.

## Results

### Samples

Between 25 October, 2010 and 23 December, 2011, a blood sample was obtained from each of 2,585 non-pregnant individuals attending the Sittwe and Thetkalpyin clinics in Rakhine State and suspected to have malaria infection. Baseline characteristics of the included population are shown in Table 3.

**Table 3:** Baseline characteristics of the study population

	
**Male**	1,327 (51.3)
**Mean age in years (range)**	10.9 (0.1-94)
**Mean temperature in °C (range)**	39.2 (35.0-42.0)
**Temperature ≥37.5°C**	2,162 (85.0)
**Parasitaemia level -**	1,337 (51.7)
**Parasitaemia level +**	161 (6.2)
**Parasitaemia level ++**	132 (5.1)
**Parasitaemia level +++**	340 (13.2)
**Parasitaemia level ++++**	615 (23.8)

The included population for the study was all non-pregnant patients presenting at the Sittwe and Thetkalpyin clinics, Rakhine State, Myanmar with fever or a history of fever prior 24 hours.

Data are presented as numbers (%) unless otherwise indicated.

Microscopy and RDT results are shown in Table 1: 531/2,585 (20.5%) patients were infected with *P. falciparum*, 587/2,585 (22.7%) with *P. vivax or P. malariae*. and 1,337/2,585 (51.7%) tested negative; 130 (5.0%) mixed infections (i.e., six from Sittwe, 124 from Thetkalpyin) were found and excluded from the main analysis, only misclassification was assessed

### Test performance

Sensitivity, specificity and predictive values of the SD 05FK60 for detection of malaria are shown in Table 2. The overall sensitivity for *P. falciparum* detection by the SD 05FK60 was 90.2% (95% CI: 87.4-92.6) and for *P. vivax/P. malariae* 79.4% (95% CI: 75.9-82.6). The overall specificity for *P. falciparum* detection by the SD 05FK60 at both clinics was 98.5% (95% CI: 97.7-99.1) and for *P. vivax/P. malariae* 98.7% (95% CI: 97.9-99.2).

The positive predictive value (PPV) for *P. falciparum* detection by the SD 05FK60 in both projects was 96.0% (95% CI: 93.9-97.5%) and for *P. vivax* 96.3% (95% CI: 94.2-97.8%) at prevalence levels of 28% and 31%, respectively. The negative predictive value (NPV) for *P. falciparum* detection by the SD 05FK60 in both projects was 96.3% (95% CI: 94.9-97.5%) and for *P. vivax* 92.5% (95% CI: 90.7-94.2%). Test accuracy indices were calculated separately for the two clinics but no significant difference in performance was observed.

2.5% (13/531) of the *P. falciparum* infections were wrongly identified by the SD 05FK60 as non-falciparum infections (Table 1). Of those misclassified two samples had a +, one sample a ++, four samples a +++ and six samples a ++++ parasitaemia level.

Of the non-falciparum infections 2.5% (15/587) were misclassified as *P. falciparum* or mixed infection (Table 1): 0.5% (3/587) were positive on the *P. falciparum* band of which one samples had a ++ and one sample ++++ parasitaemia level. 2.0% (12/587) of the non-falciparum samples were positive on the *P. falciparum* and pan-band of which seven had a +++ and five ++++ parasitaemia level. Of the 130 mixed infections 122 were correctly identified by the SD 05FK60 with a positive *P. falciparum* and pan-band. Two samples only had a positive pan-band and six samples were only positive on the *P. falciparum* band.

Table 4 shows the malaria detection rate with SD 05FK60 at different levels of parasitaemia. The sensitivity varied between 90.5% and 95.8% for *P. falciparum* specimens and between 69.8% and 97.7% with a ++ to ++++ parasitaemia level. The sensitivity increased consistently with increasing parasitaemia for *P. vivax/P. malariae* (p < 0.005). 25/408 and 13/420 cases with +++ and ++++ parasitaemia levels, respectively, of *P. falciparum* and non-falciparum malaria were missed by the RDT.

**Table 4:** Sensitivity by parasitaemia level of the SD 05FK60 rapid diagnostic test

**Parasite density**	***P. falciparum***		***P. vivax/P. malariae***	
	**Number of RDT positives/Total positives by micoscopy**	**Sensitivity %**	**Number of RDT positives/Total positives by micoscopy**	**Sensitivity %**
+	34/57	59.7	14/104	13.5
++	62/66	93.9	44/63	69.8
+++	134/148	90.5	153/159	96.2
++++	249/260	95.8	255/261	97.7
Total	479/531	90.2	466/587	79.4
Chi-square test for trend	p <0.05		p <0.05	

A total of 2,585 blood samples was examined at the Sittwe and Thetkalpyin clinics, Rakhine State, Myanmar.

Estimated parasite densities: + = 10–90 p/μl, ++ = 100–1,000 p/μl, +++ = 1,000-10,000 p/μl, ++++ = >10,000 p/μl. N: total number of blood samples; n: total detected by SD Bioline Malaria Ag P.f/Pan (05FK60).

### Quality control

The quality control team at the Shoklo Malaria Research Unit (SMRU) in Mae Sot, Thailand, cross-checked 450 slides (225 negative and 225 positive) from MSF’s projects in Myanmar from the year 2010, and 250 slides (125 positives and 125 negatives) from MSF’s projects in Eastern Rakhine State in Myanmar from the year 2011.

The overall agreement between the positive results had a kappa value of 0.96 (95% CI: 0.93; 0.99) for 2010 and 0.91 (95% CI: 0.85, 0.98) for 2011, indicating very good agreement. The overall sensitivity was 98.3% (225/229) in 2010 and 98.4% (123/125) in 2011; overall specificity was 100% (211/211) in 2010 and 99.1% (110/111) in 2011.

## Discussion

Commercially available HRP-2/pLDH tests have been evaluated previously in Myanmar [29,30] but evaluations of the SD 05FK60 are available only from other areas and were conducted before 2007 [17-22]. The study showed overall good performance for the SD 05FK60 RDT in the identification of malaria infections caused by *P. falciparum* and satisfactory results for non-falciparum infections. The poorer detection and identification of *P. vivax/P. malariae* than that of *P. falciparum* is consistent with other evaluations of the SD 05FK60 and pan-pLDH tests in general [17-22].

However, it should be pointed out that in Round 1 of the WHO/TDR/FIND/CDC evaluation, the panel detection score (PDS) of the SD 05FK60 reported at parasitaemia levels of 200 parasites/μl was 96.2% for *P. falciparum* and 35.0% for *P. vivax*[18]*.* The same product was retested in Round 3 of this evaluation and showed a slightly decreased PDS of 92.9% for *P. falciparum* along with an improved detection score of 97.1% for *P. vivax*[19]. It is unclear which version of the product was received in the programme - the catalogue number always remained the same – and this inconsistency is a commonly encountered problem by MSF when an *in vitro* diagnostic product is changed.

The sensitivity of the SD 05FK60 improves with increasing parasitaemia. This is a well-known limitation of RDTs. Less expected were the findings that 25 *P. falciparum* infections and 12 *P. vivax/P. malariae* infections with +++ or ++++ parasitaemia were undetected by the SD 05FK60. The non-detection of *P. falciparum* infections at high parasitaemia levels could be explained by the prozone effect [31-33] or by deletion of the histidine-rich repeat region in the HRP-2 gene [34]. It was not possible to conduct further molecular analysis of the *Plasmodium* cohort in the area to undermine this argument. However, this does not explain the false negative pan-pLDH band.

Translating these results into the impact on patients, 52 (10.2%) of a total 531 *P. falciparum* mono-infections would have been missed by using the RDT without microscopy. Although the specificity has been high for both *P. falciparum* and non-falciparum infections: 98.5% and 98.7% respectively, thirteen infections were misidentified as *P. vivax/P. malariae* malaria and as a result would have been treated with chloroquine, a drug to which *P. falciparum* is unlikely to be susceptible [35]. Considering the *P. vivax/P. malariae* cases, 121 out of 587 (20.6%) individuals would have not received any malaria treatment despite being infected. Only 31 individuals out of a total of 1,337 (2.3%) would have been unnecessarily treated with anti-malarials on the basis of RDT.

Although the results of the performance of the SD 05FK60 are satisfactory, MSF decided to opt for microscopy as the first choice for malaria diagnosis in its programmes in Rakhine State, Myanmar. This was a pragmatic decision based on the clinicians’ greater trust in microscopy, particularly in light of the cases with high parasitaemia that would have been missed if microscopy had not been used in addition to the RDT. MSF reserves RDTs for use in decentralized settings and where a high number of patients precludes microscopy. Therefore MSF decided to continue using microscopy in the two clinics in Rakhine State, Myanmar for the routine diagnosis of malaria, although RDTs have been found to be more cost-effective and more feasible in other settings [36-38].

This study has several limitations. The data were collected as part of routine programmatic activities and were unlinked to patient records. Thus, it was impossible to collect clinical information, such as recent intake of anti-malarials or the presence of other factors, which may have influenced the results. Another limitations is that the + system has been used to grade parasitaemia instead of a parasite count. This less accurate estimation may have influenced the results on sensitivity especially in the stratified analysis of parasitaemia levels.

It is important to note that the described data were obtained in true field conditions, i e, conditions that were suboptimal in terms of RDT storage and handling and staff expertise, and non-standardized specimens may have been present. It is known that such factors influence RDT performance [39,40], but it is difficult to optimize all these variables under difficult real-life conditions. However, this is also a strength of the study, as it represents test performance under field conditions.

MSF is able to train and maintain good malaria microscopy in its field settings. However, this may not be possible in a wider public health arena. In 2006 the WHO estimated that, if the recommendation to obtain parasitological confirmation of malaria before prescribing treatment were to be followed, screening would need to be scaled up from 122 million to 780 million tests each year in Southeast Asia alone, a 600% increase. Such scaling up would undoubtedly be difficult to achieve without the use of simple, reliable RDTs [7].

## Competing interests

The authors declare that they have no competing interests.

## Authors’ contributions

WTN organized the sample collection and quality control in Rakhine State and performed the data entry. CSK performed the statistical analysis. CSK, WTN, EP and LS analysed and interpreted the results and drafted the manuscript. All authors approved the final manuscript.
